# Exploring the host response in infected lung organoids using NanoString technology: A statistical analysis of gene expression data

**DOI:** 10.1371/journal.pone.0308849

**Published:** 2024-11-26

**Authors:** Mostafa Rezapour, Stephen J. Walker, David A. Ornelles, Muhammad Khalid Khan Niazi, Patrick M. McNutt, Anthony Atala, Metin Nafi Gurcan

**Affiliations:** 1 Center for Artificial Intelligence Research, Wake Forest University School of Medicine, Winston-Salem, NC, United States of America; 2 Wake Forest Institute for Regenerative Medicine, Wake Forest University School of Medicine, Winston-Salem, NC, United States of America; 3 Department of Microbiology and Immunology, Wake Forest University School of Medicine, Winston-Salem, NC, United States of America; Centers for Disease Control and Prevention, UNITED STATES OF AMERICA

## Abstract

In this study, we used a three-dimensional airway “organ tissue equivalent” (OTE) model at an air-liquid interface (ALI) to mimic human airways. We investigated the effects of three viruses (Influenza A virus (IAV), Human metapneumovirus (MPV), and Parainfluenza virus type 3 (PIV3) on this model, incorporating various control conditions for data integrity. Our primary objective was to assess gene expression using the NanoString platform in OTE models infected with these viruses at 24- and 72-hour intervals, focusing on 773 specific genes. To enhance the comprehensiveness of our analysis, we introduced a novel algorithm, namely MAS (Magnitude-Altitude Score). This innovative approach uniquely combines biological significance, as indicated by fold changes in gene expression, with statistical rigor, as represented by adjusted p-values. By incorporating both dimensions, MAS ensures that the genes identified as differentially expressed are not mere statistical artifacts but hold genuine biological relevance, providing a more holistic understanding of the airway tissue response to viral infections. Our results unveiled distinct patterns of gene expression in response to viral infections. At 24 hours post-IAV infection, a robust interferon-stimulated gene (ISG) response was evident, marked by the upregulation of key genes including IFIT2, RSAD2, IFIT3, IFNL1, IFIT1, IFNB1, ISG15, OAS2, OASL, and MX1, collectively highlighting a formidable antiviral defense. MPV infection at the same time point displayed a dual innate and adaptive immune response, with highly expressed ISGs, immune cell recruitment signaled by CXCL10, and early adaptive immune engagement indicated by TXK and CD79A. In contrast, PIV3 infection at 24 hours triggered a transcriptional response dominated by ISGs, active immune cell recruitment through CXCL10, and inflammation modulation through OSM. The picture evolved at 72 hours post-infection. For IAV, ISGs and immune responses persisted, suggesting a sustained impact. MPV infection at this time point showed a shift towards IL17A and genes related to cellular signaling and immune responses, indicating adaptation to the viral challenge over time. In the case of PIV3, the transcriptional response remained interferon-centric, indicating a mature antiviral state. Our analysis underscored the pivotal role of ISGs across all infections and time points, emphasizing their universal significance in antiviral defense. Temporal shifts in gene expression indicative of adaptation and fine-tuning of the immune response. Additionally, the identification of shared and unique genes unveiled host-specific responses to specific pathogens. IAV exerted a sustained impact on genes from the initial 24 hours, while PIV3 displayed a delayed yet substantial genomic response, suggestive of a gradual and nuanced strategy.

## 1. Introduction

The human bronchial tree is a complex and heterogeneous system, serving crucial functions beyond mere air conduction and gas exchange. It acts as a critical interface between the internal and external environments, facilitating essential processes such as mucociliary clearance, airway humidification, pathogen and particulate detection, and defense mechanisms. It also plays a signaling role to the underlying mesenchyme and immune system [1]. The airway epithelium itself is diverse, composed of specialized cells such as ciliated cells, goblet cells, club cells, basal cells, ionocytes, and neuroendocrine cells [2]. Dysfunction in these epithelial cell types correlates directly with respiratory diseases including asthma, chronic obstructive pulmonary disease (COPD), and cystic fibrosis [3].

Moreover, there is significant interaction between the pseudostratified epithelium and its three-dimensional (3D) microenvironment, which includes subepithelial fibroblasts, immune cells, endothelial cells, and smooth muscle cells, varying with the airway size. Each of these cell types within the airway interstitium plays a pivotal role in cellular communication influencing both normal function and disease pathology [4, 5]. In addition to the cellular heterogeneity, the subepithelial extracellular matrix (ECM) has shown to be vital for airway structural integrity and in regulating cellular functions such as activation, proliferation, and differentiation. Specific ECM-cell receptor signaling driven by the composition of airway collagen and its stiffness has been directly linked to various disease phenotypes and pathologically activated cells [6].

Despite advances in pulmonary medicine, current non-clinical models fail to optimally represent these complex cell–cell, ECM-cell, and biomechanical components of the human airways. The gold standard for studying human airways involves a monoculture of human bronchial epithelial (HBE) cells cultured on porous polymer membranes at an air-liquid interface (ALI). This model, while beneficial for promoting HBE differentiation and pseudostratification, and allowing functions like mucus production and ciliation, lacks physiological cell–cell interactions with non-epithelial cells and does not adequately mimic cell-ECM interactions beyond a simple collagen coating intended to resemble the basal membrane [7]. The significantly stiffer growth surface compared to native tissue also potentially alters HBE phenotype, heterogeneity, and functionality, thus not accurately mirroring the in vivo airway epithelial conditions [8–10].

Recognizing these limitations, there has been a push towards developing 3D cell culture and microfluidic models that better replicate the in vivo microenvironment. While spheroidal models offer improved cell-cell interactions and an ECM-based environment, they lack a crucial air-liquid interface, which is essential for analyzing cilia and mucus interactions along with aerosolized drug and toxin exposure. Similarly, planar microfluidic models, despite their advances, often do not recreate the biomechanical ECM environment adequately [11, 12].

To address these gaps, our group introduces a novel planar airway 3D organ tissue equivalent (OTE) model that incorporates a well-differentiated HBE layer maintained at ALI on a hydrogel substrate that can house native lung fibroblasts and solubilized human lung ECM [13]. This model facilitates comprehensive studies on the physiological cell–cell, ECM-cell, and biomechanical interactions, offering a significant advancement over existing models. The following sections will detail the development and evaluation of this innovative 3D OTE model, emphasizing its potential to revolutionize our understanding of human airway function and disease in vitro [13].

In the realm of infectious disease research, 3D human tissue-engineered models have emerged as critical tools. Rudra et al. [14]. developed a 3D Human Tissue-Engineered Lung Model (3D-HTLM) using chitosan-collagen scaffolds to mimic the human lung’s complex architecture more accurately, enhancing the cellular response to Influenza A Virus (IAV) infections compared to 2D cultures. This model demonstrated significant differences in cell viability, morphology, and cytokine release [14]. Zhou et al. [15]. used 3D human airway organoids to enhance the assessment of influenza virus infectivity, proving especially useful in evaluating the infectivity of emerging strains. Kinder et al. [16] focused on comparing infection mechanisms between Respiratory Syncytial Virus (RSV) and Human Metapneumovirus (MPV) using 3D human airway epithelial tissues, revealing distinct viral transmission strategies.

Moreover, Ribó-Molina et al. [17] applied organoid-derived bronchial culture models to study MPV, offering insights into the virus’s targeting of ciliated epithelial cells and subsequent cilia damage. Such models closely simulate human respiratory tract conditions, providing a more accurate platform for evaluating viral replication and pathogenesis. Further studies by Rijsbergen et al. [18] and Shpichka et al. [19] have underscored the efficacy of 3D organoids in studying various viral infections, including Human Parainfluenza Virus Type 3 (PIV3), highlighting their superiority over traditional 2D cultures in replicating key aspects of infections and aiding in the exploration of antiviral treatments.

Building on the foundation laid by the development and application of advanced 3D human tissue-engineered models for studying respiratory viruses, this paper pivots to a focused exploration of the responses of human airway tissues to viral infections using upper airway lung OTEs [13]. Given the significant health impact of viral respiratory infections, such as those caused by IAV, MPV, and PIV3, which contribute to substantial morbidity and mortality globally, a deeper understanding of their pathogenesis is crucial for developing effective therapeutic strategies.

This research meticulously tracks gene expression changes at 24- and 72-hours post-infection, focusing on 773 genes to highlight both unique and shared biological pathways affected by these viruses. Employing novel analytical methods, such as the Magnitude-Altitude Score (MAS) and Agreement Assessment of Common Genes with Dual Baselines, ensures that our interpretations of the data are both biologically relevant and statistically robust.

Central to our study are several pivotal questions and hypotheses aimed at unraveling the intricate gene expression dynamics induced by IAV, MPV, and PIV3. We hypothesize that each virus triggers a unique set of genetic changes due to its specific interactions with host cellular mechanisms. This is examined through rigorous differential expression analysis, aiming to identify distinct patterns of gene expression in response to each virus at different post-infection times. Additionally, we anticipate significant temporal dynamics in the gene expression response to each virus, reflecting the progression of the infection and the host’s evolving strategy to control viral replication and spread. This temporal aspect is crucial for understanding how initial immune responses transition over time to more sustained or regulatory mechanisms.

Furthermore, our study probes whether there are common defense mechanisms activated by all three viruses. We posit that despite the unique responses, there will likely be a core set of genes or pathways consistently activated across all infections as a universal defense mechanism. This insight is pivotal for identifying potential targets for broad-spectrum antiviral therapies.

The contributions of these findings to our understanding of viral pathogenesis are manifold. Firstly, the identification of virus-specific genomic signatures provides deep insights into how each virus interacts with the host. Secondly, the insights into the temporal dynamics of host responses are invaluable. Understanding when certain genes are upregulated or downregulated can help in pinpointing the critical periods of infection when therapeutic interventions could be most effective. Additionally, our study reveals the complexity of the immune response, which does not merely act in a linear fashion but adapts and evolves over the course of the infection.

Overall, this study significantly advances our knowledge of how respiratory viruses interact with 3D airway OTEs. By mapping the intricate patterns of gene expression in response to viral infections, it offers a foundation for future research aimed at developing effective strategies to manage and treat these infections. Understanding these interactions in greater detail will be crucial for the development of targeted therapies and for improving our overall response to viral pathogens.

## 2. Materials and methods

### 2.1. Data

Our group has developed a 3D airway “organ tissue equivalent” (OTE) model, situated at an air-liquid interface (ALI), to mimic the complexities of human airways, involving cellular interactions, extracellular matrix (ECM) proteins, and biomechanical factors [13]. The 3D organ tissue equivalent (OTE) model for studying human airway epithelium employs primary human bronchial epithelial cells (HBEs) sourced from a single donor (identified as DD057o). These HBEs were cultured at an air-liquid interface to promote differentiation and functionality. Alongside HBEs, native human lung fibroblasts (sourced from donor 0000543644) were integrated into the hydrogel matrix to mimic the stromal environment of the lung. This incorporation enhances the model’s physiological relevance by facilitating direct cell-cell interactions and providing a more realistic microenvironment [13].

The model utilizes cells from individual donors to establish the baseline behavior of the airway epithelium in a controlled setting. This approach allows for the assessment of typical cellular responses within the OTE system without the variability that might arise from using multiple genetically distinct donors. However, future studies are suggested to expand the cell source to multiple donors to address genetic diversity and its impact on the disease modeling and pharmacological studies. This expansion will be crucial for understanding varied responses to treatments across different genetic backgrounds and enhancing the model’s applicability to a broader human population [13].

The OTE model at the ALI is chosen as the experimental model for its ability to represent the complex microenvironment of the human airways more accurately. This model is designed to overcome the limitations of traditional 2D ALI models, which lack essential interactions found in vivo. Here are several key features that enable the 3D OTE model at the ALI to mimic the natural human airway environment more accurately:

- Complex Cell-Cell Interactions: Unlike 2D models that only use human bronchial epithelial (HBE) cells, the 3D OTE incorporates both solubilized extracellular matrix (ECM) from human lungs and native lung fibroblasts. This combination supports more physiological cell-cell interactions, crucial for maintaining the structural and functional integrity of the epithelial barrier [13].
- ECM-Cell Interactions: The model includes a hydrogel substrate that mimics the lung’s ECM, enriched with components like collagen, elastin, and hyaluronic acid. This setup facilitates natural cell adhesion, growth, and differentiation, which are essential for accurate modeling of airway physiology [13].
- Biomechanical Properties: The hydrogel’s biomechanical properties can be adjusted to represent the range of stiffness found in healthy and diseased airway tissues. This variability is crucial for studying how mechanical forces influence cell behavior, which is particularly relevant for diseases like asthma or pulmonary fibrosis [13].
- Enhanced Differentiation at ALI: Culturing HBE cells at the ALI promotes better differentiation and functionality, including mucus production and ciliary motion, which are critical for understanding airway defense mechanisms against pathogens and particulates [13].

By integrating these elements, the 3D OTE model at the ALI offers a more dynamic and representative environment for studying the human airway, providing insights that are more applicable to in vivo conditions [13].

#### Viral infection of OTE models with IAV, MPV, and PIV3

Expanding on the utility of this model, we introduced viral agents, specifically three viruses, to infect the OTEs. We utilized three viruses to infect the OTEs as part of the infection process:

- IAV (Influenza A virus): A/Puerto Rico/8/1934 (H1N1) with an EGFP-NS1 gene fusion.
- MPV (Human metapneumovirus): This virus has the EGFP gene inserted upstream of the N gene of strain CAN97-83 (ViraTree product #M121).
- PIV3 (Human parainfluenza virus type III): This virus has the EGFP gene inserted between the first (N) and second (P/C/D/V) genes of strain JS (ViraTree product #P323).

#### Preparation and application of infection medium for viral titration in OTE models

Virus infection medium (iDMEM) was prepared as DMEM with 0.1% heat-inactivated fetal bovine serum, 0.3% purified bovine serum albumin, 20 mM HEPES [pH 7.5] and 0.2 mM Glutamax, filter-sterilized, and stored at 4°C. The surface of each OTE to be infected was gently washed by briefly adding 100 μl of warm Hank’s Balanced Saline Solution followed by gentle aspiration. Virus diluted in iDMEM was warmed to room temperature and added in a 40 μl volume the apical surface of the OTE. The plate containing the infected OTEs was placed on a rocking table in the 37°C CO_2_ incubator for one h before being removed from the rocking table and returned to normal growth conditions. Influenza virus titer was determined fluorescent-focus assay using MDCK cells (ATCC product #CCL-34). Metapneumovirus and parainfluenza virus type 3 titers were determined by fluorescent-focus assay using the LLC-MK2 derivative cell line (ATCC product #CCL-7.1) Both MDCK and LLC-MK2 cells were maintained in DMEM supplemented with 10%FBS. OTEs were infected at a nominal multiplicity of 0.1 infectious units per susceptible epithelial cell in the culture. The identical amount of UV-inactivated virus was used for infections with UV-inactivated virus.

#### Virus inactivation by ultraviolet light

Aliquots of virus stored at -80°C were thawed at room temperature and diluted into iDMEM. The diluted virus was placed in a sterile polystyrene vessel of an appropriate size to ensure that the depth of the virus in solution was 2 mm or less. Samples were placed in a UV crosslinker (Spectroline model XLE-1000) and exposed to 4 mW per cm^2^ of UV-C (254 nm) irradiation for 240 seconds. This amount of radiation was determined to be approximately 8-times longer than the exposure required to achieve complete inactivation of influenza virus. The inactivated virus sample was immediately used for infection or distributed among sterile microcentrifuge tubes and stored at -80°C until use.

#### NanoString analysis of viral infections in OTE models

We employed the NanoString nCounter® Analysis System for highly multiplexed detection of mRNA targets in our study of viral infections within OTE models. Utilizing the nCounter® Host Response Panel (Cat# XT-HHR-12), which includes probes for 785 human genes and 12 internal reference genes, we analyzed various host responses, such as susceptibility, interferon signaling, and immune cell activation.

#### Control strategies in viral infection of OTEs for enhanced reliability

In our study, we implemented various control conditions to enhance the precision of our experimental outcomes and effectively distinguish the effects of viral infection from other experimental variables. These control conditions, including mock-infected, UV-treated, and naïve samples, are pivotal for affirming the integrity and reliability of our findings.

Mock-infected samples serve as the primary baseline for comparison with actively infected samples. These samples undergo all experimental procedures without the introduction of viruses, ensuring that any observed gene expression changes can be attributed to the infection rather than the experimental process itself. This baseline is crucial for conducting pairwise multiple hypothesis testing to identify gene expression changes that are a direct consequence of active viral infection, offering precise insights into virus-host interactions.

UV-treated samples are subjected to ultraviolet light to inactivate any present viruses, serving as a critical control for understanding the impact of viral components without their active replication. By using these samples, we can isolate the effects of viral entry and subsequent host cell responses from those induced by viral replication. This approach helps to specifically identify how viral infection mechanisms influence gene expression changes, separate from the effects of active viral proliferation within host cells. The primary role of UV-treated controls is to understand the structural and molecular components of viruses that can still interact with host cells without active viral replication, delineating the effects solely attributable to the viral entry and initial interactions with the host cells from those caused by viral replication.

Naïve or untreated samples, referred to as untreated OTEs, serve as internal controls to establish a gene expression baseline across our experiments. These samples do not undergo any experimental manipulation, such as virus introduction or mock treatments. The role of naïve samples is to provide a control for natural variations in gene expression that occur without any external interventions. By comparing infected and mock-infected samples to these baselines, we ensure that observed gene expression changes are indeed due to the experimental conditions (e.g., viral infection) rather than inherent variability in the model system.

Despite the indispensable role of UV-treated and naïve samples in establishing baseline and control conditions, our differential expression analysis focused exclusively on the direct effects of active viral infection on gene expression within the OTE models. The strategic exclusion of UV-treated and naïve samples from expression analysis underscores our commitment to methodological rigor, maintaining a sharp focus on the specific impacts of viral infection.

Note that, we chose 24- and 72-hours post-infection based on preliminary studies [20–24]. These studies indicated that these time points effectively capture the characteristic changes in gene expression following viral infections. Specifically, the 24-hour mark allows us to observe the immediate, rapid responses typical of viruses like IAV, which are crucial for understanding early host-virus interactions. The 72-hour point, on the other hand, is critical for identifying more delayed responses, which are particularly relevant for viruses like PIV3 that exhibit slower disease progression.

#### Categorization of infection conditions in OTEs based on virus type, treatment, and time post-infection

We categorized the infection conditions based on the virus type (IAV, MPV, or PIV3), treatment (UV or Non-UV/active), and post-infection time (24 or 72 hours). Active viral infections were labeled as Virus-active-Time, and to establish a baseline for comparison, we included mock-infected samples (Mock-24 and Mock-72), and UV-treated samples (Virus-UV-Time). Each of these conditions was replicated six times at both the 24- and 72-hour time points, ensuring robustness and reliability in our results (see **Table 1)**. Note that there was no additional stimulation involved in these experiments. The samples were exposed to one of three conditions: infection medium containing the virus (active/ no treatment), infection medium containing the inactivated virus (UV-treated), or infection medium alone (non-stimulated control).

**Table 1:** Sixteen infection conditions categorized based on (1) Virus, (2) Treatment, and (3) post-infection time.

**Primary** **Condition**	**Virus**	**Treatment**	**Post-infection Time**	**Primary** **Condition**	**Virus**	**Treatment**	**Post-infection Time**
IAV-None-24	IAV	active/ no treatment	24-hours	IAV-None-72	IAV	active/ no treatment	72-hours
MPV-None-24	MPV	active/ no treatment	24-hours	MPV-None-72	MPV	active/ no treatment	72-hours
PIV3-None-24	PIV3	active/ no treatment	24-hours	PIV3-None-72	PIV3	active/ no treatment	72-hours
Mock-24	Mock	-	24-hours	Mock-72	Mock	-	72-hours
**Negative** **Control**	**Virus**	**Treatment**	**Post-infection Time**	**Negative** **Control**	**Virus**	**Treatment**	**Post-infection Time**
IAV-UV-24	IAV	UV	24-hours	IAV-UV-72	IAV	UV	72-hours
MPV-UV-24	MPV	UV	24-hours	MPV-UV-72	MPV	UV	72-hours
PIV3-UV-24	PIV3	UV	24-hours	PIV3-UV-72	PIV3	UV	72-hours
Untreated-24	-	-	24-hours	Untreated-72	-	-	72-hours

### 2.2. Differential expression analysis via the Magnitude-Altitude Score (MAS) algorithm

With the extensive data generated from our meticulously conducted experiments, a comprehensive analysis approach becomes paramount to uncover the intricate patterns of gene expression and draw meaningful biological conclusions. Traditional methods of differential gene expression analysis have often fallen short in this regard, predominantly focusing on statistical significance, and inadvertently sidelining the biological relevance of the genes identified. Such an approach can lead to an overemphasis on genes that, while statistically significant, may not hold substantial biological meaning in the context of viral infection and response in airway tissues.

To overcome these limitations and achieve a balanced and integrative analysis, we introduce the Magnitude-Altitude Score (MAS) algorithm into our analytical repertoire. The MAS algorithm (**Algorithm 1** in **S1 File**) is meticulously designed to ensure the identification of differentially expressed genes in genomic studies reflects genuine biological phenomena and not merely statistical artifacts. At its core, the MAS algorithm gauges biological significance by measuring the fold change, which quantifies the extent of a gene’s expression change in response to a treatment versus a control. This measure suggests the gene’s active role in responding to experimental conditions, pointing to its biological relevance. Large fold changes indicate substantial biological effects, emphasizing the gene’s potential importance in the studied process.

However, to confirm that these expression changes are not the product of random variations, the MAS algorithm integrates rigorous statistical methods. Initially, the algorithm assesses each gene’s expression variation through a p-value from a two-sample t-test (α = 0.05) [25], determining the likelihood that such changes could occur under the null hypothesis of no effect. Recognizing that p-values are prone to yielding false positives, especially in large datasets, the algorithm employs the Benjamini-Hochberg correction [26]. This correction method adjusts p-values to control the false discovery rate, greatly diminishing the risk of falsely identifying genes as significant.

The MAS algorithm synthesizes these elements by calculating a unified score for each gene, combining the magnitude of the fold change and the robustness of the statistical evidence. This score is formulated as MAS_l_= | ${({log}_{2}(\mathrm{FC}}_{l}){|}^{M}|{({log}_{10}(p}_{l}^{BH})){|}^{A}$, for l = 1, 2, …, s, where *s* is the number of rejected null hypotheses rejected based BH adjusted method ($p_{l}^{BH}<\alpha )$. M and A are hyper-parameters that balance the weight given to biological versus statistical evidence. This scoring ensures that a gene is identified as significantly differentially expressed only when it demonstrates a meaningful fold change backed by strong statistical support, thus filtering out minor fluctuations or coincidental variations in gene expression.

To further enhance reliability, the MAS algorithm ranks genes based on the MAS score that combines the magnitude of their expression changes with the adjusted p-values. By demanding that both the magnitude of expression change, and the adjusted statistical confidence exceed specified thresholds, the algorithm robustly filters out results that could be misleading due to random chance or experimental noise. The Benjamini-Hochberg adjustment process, a core component of the MAS algorithm, systematically controls the false discovery rate, essential in high-dimensional data like gene expression profiles. This adjustment ensures that the probability of declaring a gene’s expression change as significant due to random chance is tightly regulated.

Moreover, the integration of fold changes with adjusted p-values in calculating the MAS score provides a robust measure of a gene’s relevance. This ranking approach ensures that the genes identified as top differentially expressed are those with significant fold changes supported by strong statistical evidence, minimizing the impact of random variations or experimental noise. This dual-filter approach ensures that the genes highlighted are truly indicative of underlying biological processes, providing researchers with reliable and biologically meaningful insights into the molecular dynamics under study. This methodological rigor makes the MAS algorithm especially valuable in genomic research, where discerning true biological signals from a background of variability is crucial.

In summary, the MAS algorithm can be concisely represented as MAS(B,T,M,A)=(G,R,Υ,Ξ,Ρ), where *B* and *T* denote Baseline and Treated gene expressions, respectively, and *M* and *A* are hyper-parameters, *G* is the list of BH-significant genes, *R* provides their MAS ranks, Υ contains their MAS scores, Ξ includes the log_2_(FC), and Ρ contains the BH adjusted p-values.

Depending on the specific needs of the research, the hyperparameters M and A allow for the adjustment of the weightage given to fold change and adjusted p-value, respectively. In the context of our study, where the objective is to pinpoint gene fingerprints for active infection conditions within OTEs, this flexibility is invaluable. A gene with a pronounced fold change underscores a substantial biological shift between conditions, and its statistical significance ensures the result’s robustness. By setting *M* = *A* = 1 in our analysis, we ensured an equal emphasis on both these aspects, striking a harmonious balance between biological pertinence and statistical rigor.

### 2.3. The statistically significant genes that agree when a specific virus infected OTEs are contrasted against the Mock and UV infected controls

The profound complexity of host-virus interactions necessitates a robust and precise methodology to unravel the unique genomic signatures elicited by distinct viral infections. Recognizing the imperative need to differentiate the impacts of viral infection from procedural and external influences, our study introduces the “Agreement Assessment of Common Genes with Dual Baselines” algorithm (**Algorithm 2** in **S1 File**).

The primary objective of **Algorithm 2** in **S1 File** is to provide a rigorous and repeatable framework for identifying and validating the genetic impacts of viral infections, distinct from any procedural noise or external experimental influences. The process involves systematic comparisons of gene expression alterations against two control scenarios: Mock and UV-treated. This dual-baseline approach is essential as it allows us to clearly differentiate genuine viral effects from potential background noise. An integral part of this algorithm is the computation of the Agreement Ratio, which quantifies the consistency of gene expression changes across controls, further validating the impacts attributed to viral infections. This ratio is calculated as the percentage of genes that show consistent expression changes in the treated groups compared to both control groups, offering a metric to gauge the reliability of the observed changes.

By thoroughly analyzing how gene expression changes correlate across these controls, **Algorithm 2** in **S1 File** gives a clearer and more defined picture of each virus’s unique effects on gene expression within our OTE model. Such a methodical approach facilitates a deeper understanding of the specific genomic responses elicited by different respiratory viruses, critically enhancing the applicability of our findings for developing targeted therapeutic strategies.

The profound complexity of host-virus interactions necessitates a robust and precise methodology to unravel the unique genomic signatures elicited by distinct viral infections. Recognizing the imperative need to differentiate the impacts of viral infection from procedural and external influences, our study introduces the “Agreement Assessment of Common Genes with Dual Baselines” algorithm. This algorithm aims to meticulously analyze gene expression profiles from OTEs infected with different viruses, identifying unique, statistically significant genes influenced predominantly by each virus at every time point. By contrasting the results against both Mock and UV-treated controls, we enhance the reliability of our findings, ensuring that the genomic alterations observed are indeed a direct consequence of the viral infection.

### 2.4. Identifying universal and unique genomic responses to viral infections

#### Common genes selection

Understanding the common genomic responses to diverse viral infections is crucial for unraveling viral pathogenesis and developing broad-spectrum antiviral strategies. Given the dynamic and complex nature of gene expression changes elicited by viral challenges, robust computational methods are essential to identify conserved genetic signatures across different viruses and infection times. **Algorithm 3**, as detailed in **S1 File**, meets this need by employing a rigorous statistical framework to extract significant and biologically relevant common genes affected by various viral infections. This algorithm systematically compares gene expression data from OTEs infected with distinct viruses against a control baseline (Mock) at multiple post-infection time points.

The objective of **Algorithm 3** in **S1 File** is to highlight shared genetic responses that are consistently statistically significant, regardless of the virus type. By analyzing how gene expressions change in response to different viral infections compared to uninfected controls, the algorithm aims to uncover genes that are commonly affected across various infections. This process involves aggregating and analyzing data from each virus-infected group and comparing these findings to baseline control data (Mock samples). The outcome is a robust set of genes that show common alterations, thus providing valuable insights into the shared pathways that viruses exploit and how they might be targeted with therapeutic strategies. This approach not only aids in identifying potential targets for treatment but also enhances our understanding of the mechanisms of viral infection and the host’s response, which is critical for developing effective antiviral therapies.

#### Network-based gene correlation analysis

Let us assume we possess the gene expression data for *q* treated groups of OTEs, where each group is actively infected with a distinct virus, in addition to a baseline group (Mock), across various post-infection times *T*_1_,*T*_2_,…, *T*_t_. Suppose there are *q* viruses used for infection.

To evaluate the interconnections among the genes in MAS-Common-genes-*T*_i_, each gene is represented as a vector, comprising its expression levels across various conditions: Baseline_*Ti*_, and ${Treated}_{T_{i}}^{X}\;for\;X=1,2,\ldots ,q.$ Subsequently, we employ the “Network-Based Gene Correlation Analysis” algorithm (**Algorithm 4** in **S1 File**) to meticulously analyze and quantify their correlation, providing a comprehensive insight into the complex web of gene interactions under the studied conditions. This approach not only aids in understanding the coordinated expression patterns but also unveils potential regulatory mechanisms shared among the MAS-Common-genes-*T_i_*

For the setup of **Algorithm 4** in **S1 File**, we first organize the gene expression data from both baseline and various virus-treated groups into a structured format suitable for detailed analysis. Each gene’s expression levels across different conditions, baseline and treated, are compiled into vectors. These vectors are critical as they represent the expression dynamics of each gene under different experimental scenarios, thus forming the foundation for subsequent analytical steps.

In the first step of **Algorithm 4** in **S1 File**, we compute the Spearman correlation matrix [27] for all genes in the dataset. This involves calculating pairwise Spearman correlations to assess the strength and significance of the relationships between each pair of genes. To focus our analysis on the most influential gene interactions, we retain only those correlations that exceed a predefined threshold in magnitude, ensuring that we capture strong and potentially biologically significant interactions while discarding weaker and less relevant ones.

Following the correlation analysis, we construct a network where each node represents a gene, and edges represent significant correlations between these genes. This network construction is pivotal as it visualizes the intricate relationships and dependencies among genes, highlighting how they may co-regulate or influence each other’s expression in response to viral infections.

In the final step, we refine our network by focusing on genes that exhibit a high degree of connectivity. Specifically, we calculate the degree (the number of connections) for each gene and filter out those with a degree less than a set threshold. This selection criterion ensures that our final network includes only those genes with substantial interactions, thereby likely playing significant roles in the genetic response to the viruses studied.

The output of **Algorithm 4** in **S1 File** is a visual representation of the gene network, which not only aids in understanding the coordinated expression patterns among the genes but also unveils potential regulatory mechanisms shared among them. This network model provides valuable insights into how genes collectively respond to viral infections, offering potential targets for therapeutic intervention and enhancing our understanding of disease mechanisms at the molecular level.

#### Unique gene selection for a particular virus

Understanding the unique genomic signatures elicited by distinct viral infections in OTEs is crucial for identifying virus-specific therapeutic targets and unraveling the complex dynamics of host-virus interactions. While identifying common responses across viruses sheds light on shared mechanisms of viral pathogenesis, it is equally important to dissect the unique genomic alterations induced by each virus to fully grasp their specific biological impacts.

To this end, **Algorithm 5**, as detailed in **S1 File**, is crafted to meticulously analyze gene expression profiles from OTEs that have been infected with various viruses. This algorithm focuses on isolating the unique, statistically significant genes that are predominantly influenced by each virus at each observed time point. Its design allows for a precise identification of the distinct changes in gene expression that are directly attributable to specific viral infections.

The functionality of **Algorithm 5** in **S1 File** involves a thorough analysis where gene expression data from infected OTEs are compared to baseline controls to determine which genes are uniquely affected by each virus. This not only helps in pinpointing the specific genomic responses to individual viral infections but also aids in understanding how these unique responses could potentially be targeted with therapeutic strategies. By isolating these unique genes, the algorithm enhances our ability to develop targeted treatments that are finely tuned to the specificities of each viral infection, thereby advancing our capabilities in managing and treating viral diseases with precision.

## 3. Results

**Fig 1:**
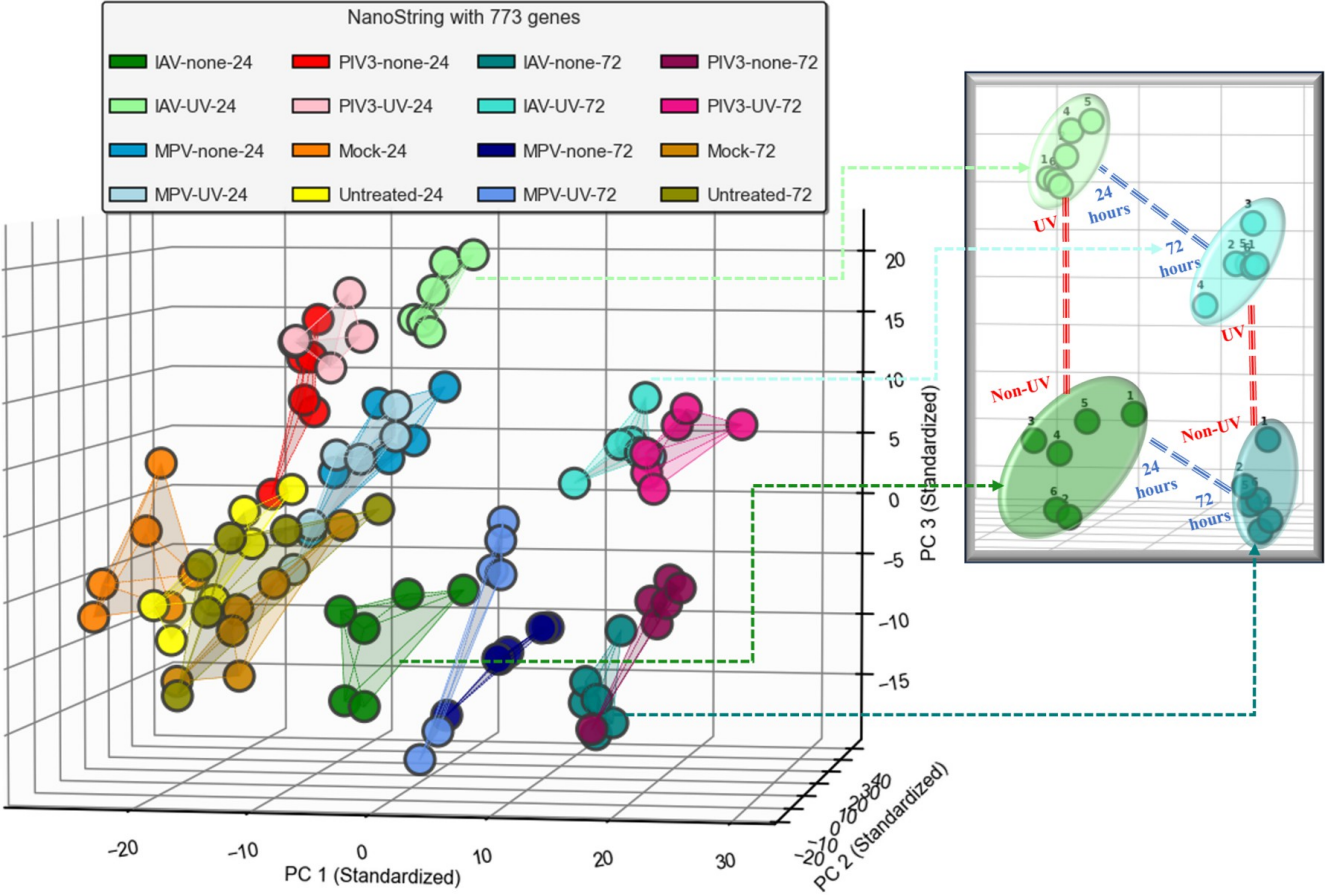
PCA-based visualization of all 96 OTEs.

In this section, we present the results obtained from applying the methods described in Section 2. For a deeper comprehension, we begin by visualizing all OTEs using PCA [28]. Given that NanoString data comprises genes with varying scales, we z-score standardize [29] the data to convert these scales to a uniform scale for a better visualization. **Fig 1** shows the projection of all OTEs (96 across all infection conditions) onto a 3-dimensional subspace created by the first three principal components. We also emphasized the distinct correlation between UV and active IAV-infected OTEs at times 24 and 72.

### 3.1. Differential expression analysis via the Magnitude-Altitude Score (MAS) algorithm

**Fig 2:**
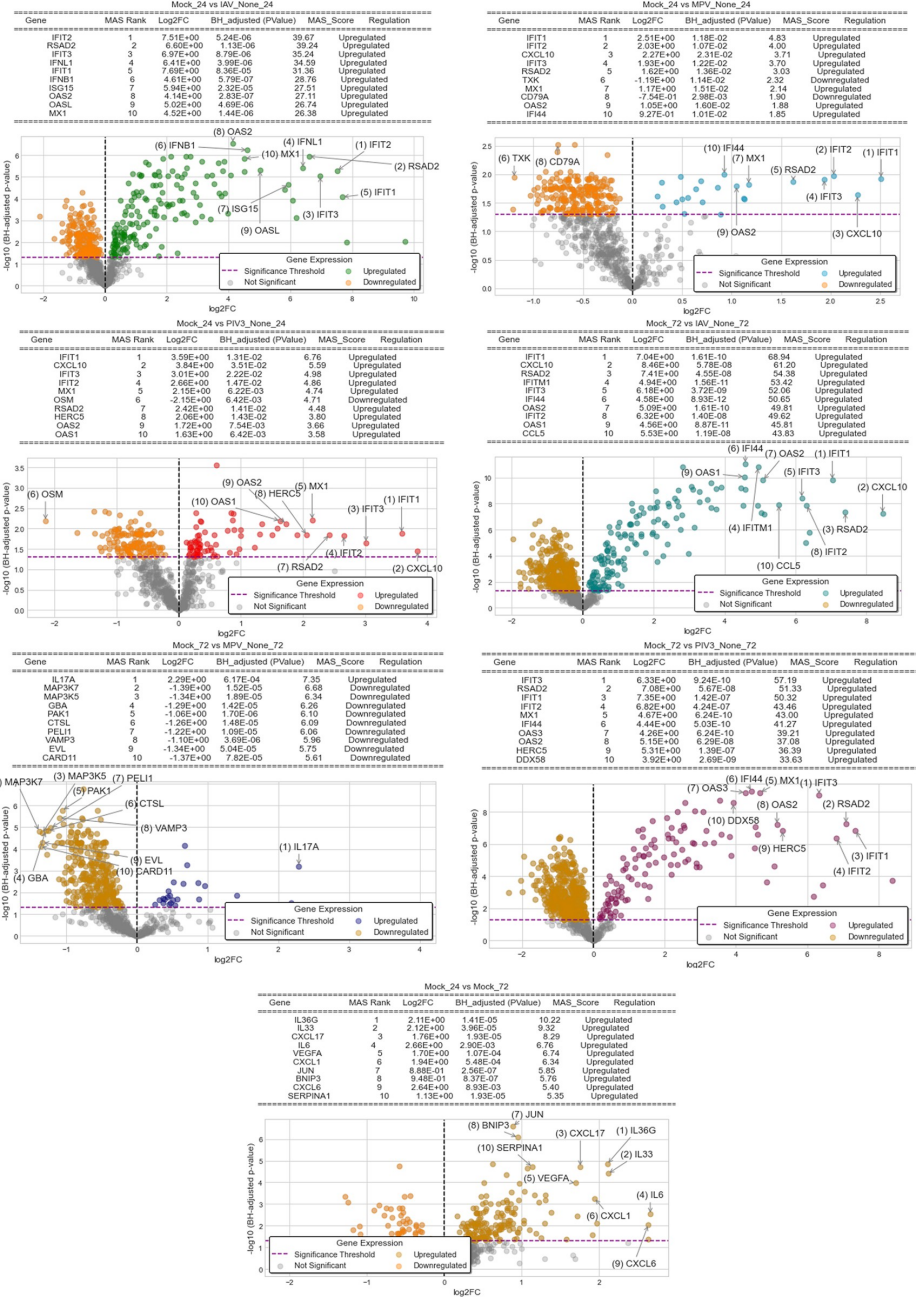
Volcano plots displaying BH-significant genes identified using Algorithm 1 in *S1 File*, with Mock-24/72 as the baseline and IAV/MPV/PIV3-24/72 as treated groups. The top 10 MAS-ranked genes for each contrast are highlighted.

Leveraging the foundational **Algorithm 1** in **S1 File**, the MAS method, we embarked on a journey to identify and rank the BH-significant genes that demarcate between varying infection conditions. Using Mock-24/72 as our control baseline and the actively infected OTEs IAV/MPV/PIV3-24/72 as our treated groups, we were able to discern patterns and hierarchies among genes in their response to these infections. The visual representation of this intricate interplay can be seen in **Fig 2**, where the volcano plots not only provide an overarching view of the differentially expressed genes but also spotlight the top 10 MAS-ranked genes for each distinct contrast.

### 3.2. The statistically significant genes that agree when a specific virus infected OTEs are contrasted against the Mock and UV infected controls

**Fig 3:**
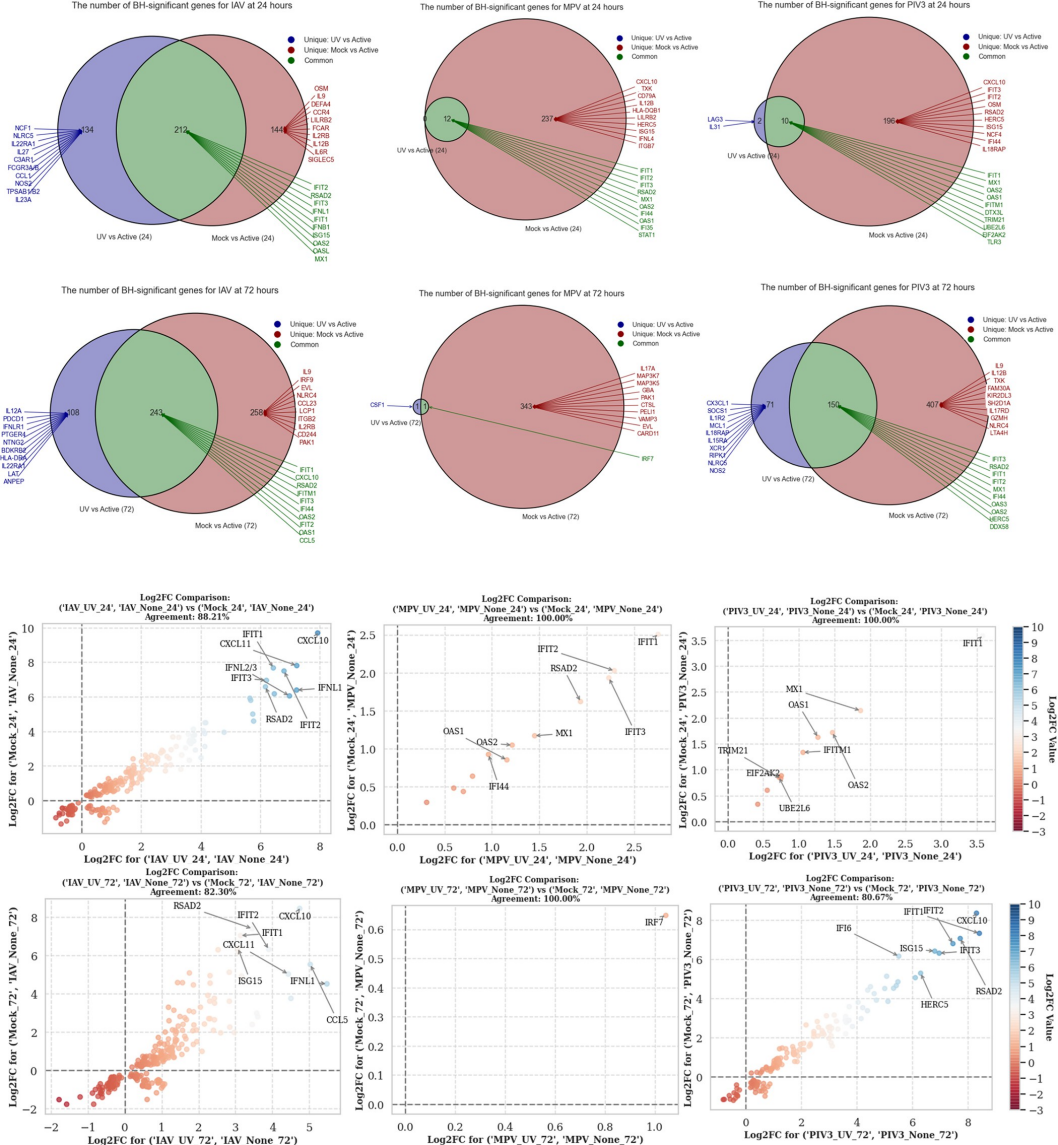
Comparative analysis of BH-significant genes using Algorithm 1 in *S1 File* with different baselines: Mock-infected and UV-infected groups. **(a)** Display of shared and unique BH-significant genes between (Mock vs. active) and (UV-infected vs. active-infected) contrasts, along with the top 10 MAS-selected genes for all six comparisons. **(b)** Representation of the consistency among shared BH-significant genes in relation to the sign of their Log Fold Change.

To further diversify our analysis, we introduced UV-infected groups as an alternative baseline, effectively juxtaposing them against the Mock-infected groups. Applying **Algorithm 2** in **S1 File**, **Fig 3A** presents a union of shared BH-significant genes across both baselines (Mock and UV-infected) in comparison to the active infection. This elucidates the unique genes and highlights the top 10 MAS-prioritized genes across the six infection contrasts. Meanwhile, **Fig 3B** provides a deeper dive into these shared BH-significant genes, illuminating the agreement ratio (AR) in their expression trends and spotlighting the directionality of their Log Fold Change.

### 3.3. Identifying universal and unique genomic responses to viral infections

**Fig 4:**
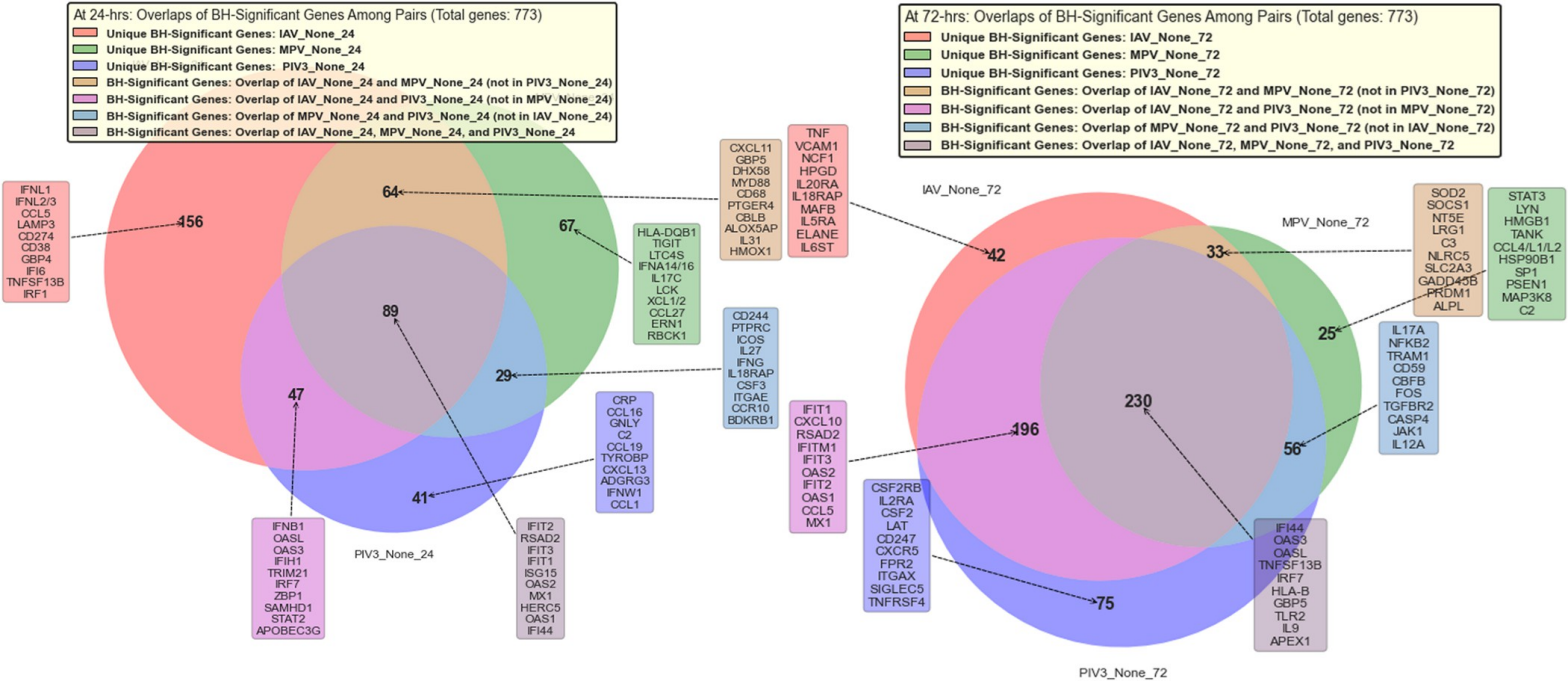
Application of Algorithm 2 in *S1 File* to identify three distinctive genes pivotal for classification.

Using Mock-infected samples as the baseline and actively infected OTEs as treated groups, **Fig 4** applies **Algorithm 3** in **S1 File** to present a Venn diagram. This diagram illustrates the significant and biologically relevant common genes that are affected by various viral infections.

**Fig 5:**
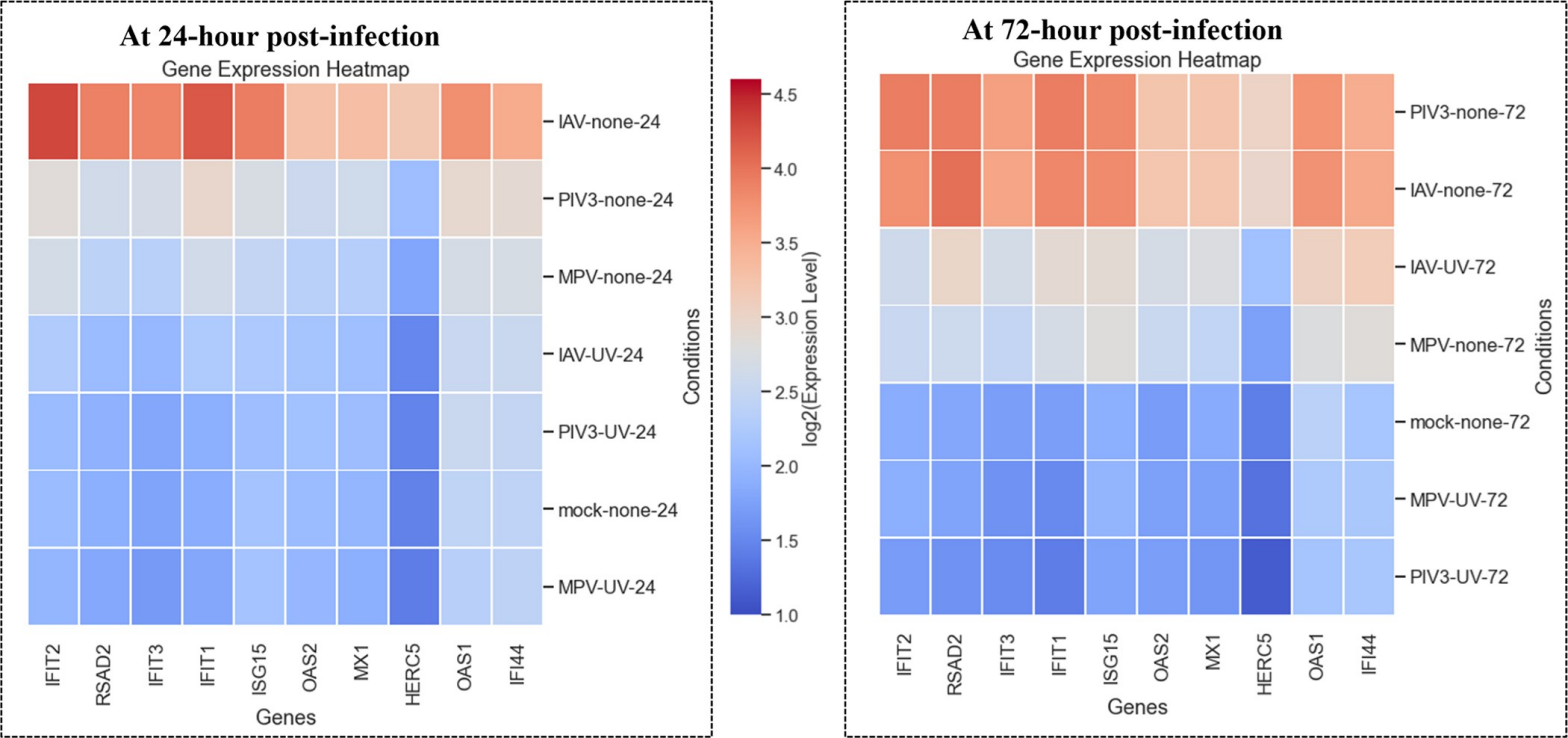
Heatmap representation of log2-transformed expression levels for the top 10 genes from MAS-Common-genes-24, measured at 24- and 72-hours post-infection. The conditions are ordered vertically by descending average expression of these selected genes.

**Fig 5** depicts the log2-transformed expression of the top 10 genes from MAS-Common-genes-24 at both 24- and 72-hours post-infection. The order of the conditions is determined based on the average expression of these genes, such that the condition with the highest average expression is positioned at the top. It turns out that, unlike with IAV and PIV3, these genes do not exhibit significant expression at 72 hours post-infection after the OTEs have been infected with MPV.

**Fig 6:**
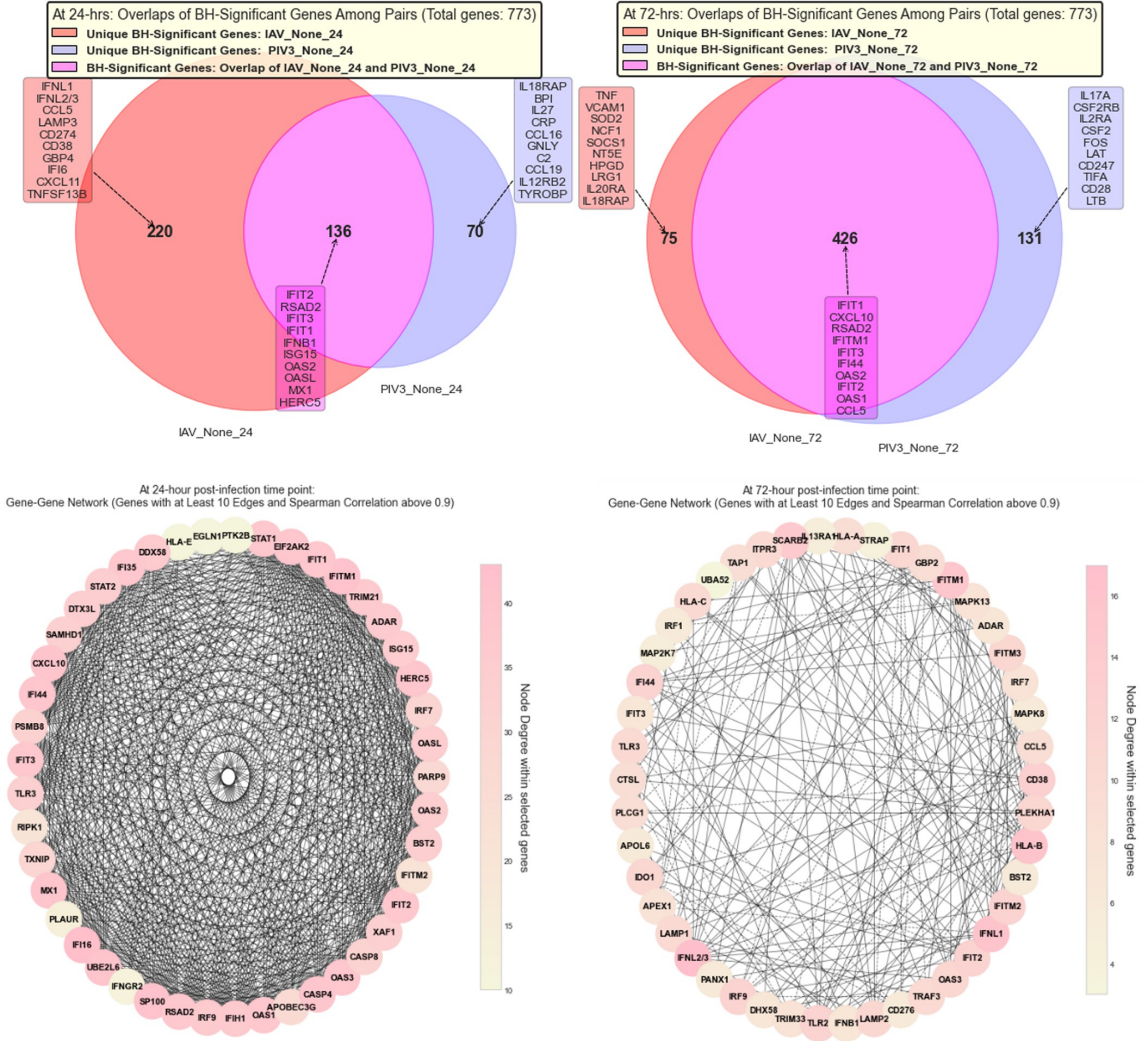
Comparative analysis of MAS-selected genes following IAV and PIV3 infections at 24- and 72-hours post-infection. Panel (a) showcases the top 10 common and unique genes, while panels (b-c) illustrate gene correlation networks at both time points with significant Spearman correlations. The color bar represents connectivity degree within the selected genes.

To gain a clearer understanding of the unique and common BH-significant genes following IAV and PIV3 infections, we excluded the OTEs infected with MPV. **Fig 6A** displays the top 10 MAS-selected genes that are common and unique at 24- and 72-hours post-infection for these two viruses. **Fig 6B–6C** displays the gene correlation networks for MAS-Common-Genes, considering only IAV and PIV3, at two post-infection time points, generated using **Algorithm 4** in **S1 File**. In **Fig 6B**, the network at *T*_1_ = 24 hours is depicted, where nodes represent genes, and edges indicate significant Spearman correlations between their expression levels, adhering to a correlation threshold of 0.9 and a minimum connectivity degree of 10. **Fig 6C** transitions to *T*_2_ = 72 hours post-infection, maintaining the same correlation and degree parameters for network construction. Note that the color bar indicates the degree of connectivity within the selected genes, not across the entire set of genes in MAS-Common-Genes.

There are a total of 136 BH-significant genes common between IAV-active-24 and PIV3-active-24 when contrasted against Mock-24. Out of these, 45 genes passed the criteria with at least 10 edges having a Spearman correlation above 0.9. The network metrics [30] are as follows: Density is 0.74, and Average Degree is 32.62. Notably, IFITM1, IFI16, and SP100 have high Betweenness and Closeness Centralities, reflecting their significance in the network. Of the 426 genes in the BH-significant genes common between IAV-active-72 and PIV3-active-72 when contrasted against Mock-72 data set, 46 passed the criteria of having at least 10 edges with a Spearman correlation above 0.9. Network Metrics: Network Density is 0.210, and Average Degree is 9.435. Node Centrality Metrics: The top 5 genes by Betweenness Centrality are CD38, IFIT1, MAPK13, IRF9, and IDO1. The top 5 genes by Closeness Centrality are IFNL2/3, CD38, IFITM1, HLA-B, and IFNL1. The top 5 genes by Eigenvector Centrality are IFNL2/3, IFITM1, HLA-B, IFNL1, and IFITM3.

Using Mock-infected samples as the baseline and actively infected OTEs as treated groups, and applying **Algorithm 5** in **S1 File**, **Fig 4** also presents the unique genomic signatures elicited by distinct viral infections in OTEs.

**Fig 7:**
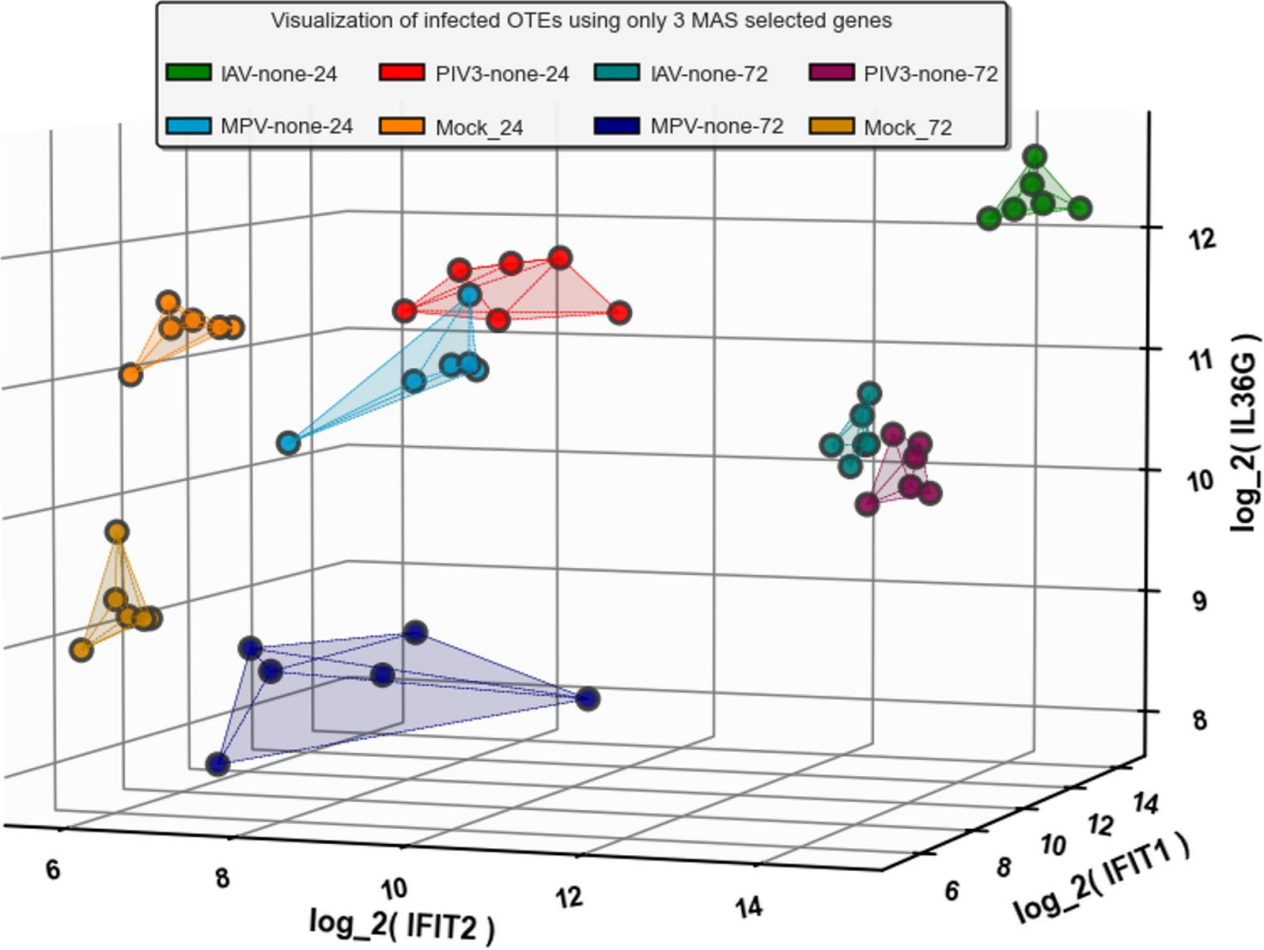
Log2-transformed expression of IFIT2, IFIT1, and IL36G, highlighting significant commonalities between IAV and PIV3 at 24 and 72 hours, and showcasing IL36G’s time-specific expression (see Fig 2 for contrast with Mock samples).

**Fig 7** illustrates the log2-transformed expression of three genes: IFIT2, IFIT1, and IL36G. IFIT2 is highlighted as the top MAS-selected gene that is significantly common between IAV and PIV3 at 24 hours, and IFIT1 is similarly noted at 72 hours. IL36G stands out as the top MAS-selected gene associated only with time, rather than infection, as demonstrated by contrasting Mock-72 against Mock-24 (see the last volcano plot in **Fig 2** for further details).

## 4. Discussion

**Fig 1** implies that there is a discernible pattern between active-infected and UV-infected OTEs at post-infection times of 24 and 72 hours, after standardizing the data. This pattern is particularly pronounced for IAV, moderate for PIV3, and weak for MPV. This discrepancy can be attributed to the fact that IAV significantly affects most of the genes within the first 24 hours post-infection, unlike MPV. On the other hand, PIV3 influences genes between 24 and 72 hours.

### 4.1. Differential expression analysis via the Magnitude-Altitude Score (MAS) algorithm

**Fig 2** provides a comprehensive visual representation of the transcriptional changes in OTEs in response to various viral infections, using volcano plots to emphasize the top differentially expressed genes as identified by the Magnitude-Altitude Score (MAS) algorithm. In the context of IAV infection at 24 hours post-infection, the MAS algorithm has identified a strong interferon-stimulated gene (ISG) response. IFIT2, RSAD2, IFIT3, IFNL1, IFIT1, IFNB1, ISG15, OAS2, OASL, and MX1 are all significantly upregulated, painting a picture of an orchestrated and robust antiviral response within the airway tissues. The presence of multiple members from the IFIT family, along with MX1, underscores the cells’ attempts to inhibit viral replication and spread. RSAD2 and the OAS family play pivotal roles in fortifying the cell’s antiviral state, actively participating in the degradation of viral components and facilitating various other antiviral activities.

Upon MPV infection at 24 hours, the top differentially expressed genes reveal a nuanced response, integrating both innate and adaptive immune signals. The ISGs IFIT1, IFIT2, IFIT3, RSAD2, MX1, and OAS2 are highly expressed, maintaining a strong antiviral state. Additionally, CXCL10 is upregulated, likely contributing to the recruitment of immune cells to the site of infection. Interestingly, this response also includes TXK, a tyrosine kinase associated with B-cell receptor signaling, and CD79A, a B-cell marker, suggesting an early involvement of adaptive immune components. IFI44, another ISG, further underlines the antiviral environment.

For PIV3 infection at 24 hours, the transcriptional response is dominated by ISGs, including IFIT1, IFIT2, IFIT3, MX1, and OAS2, indicating a potent antiviral state. CXCL10 is also upregulated, suggesting active recruitment of immune cells. The presence of OSM points towards a role in modulating inflammation and potentially contributing to antiviral defenses. RSAD2 and HERC5, both with known antiviral activities, are upregulated, highlighting the comprehensive nature of the cellular response to PIV3 infection.

By 72 hours post-IAV infection, the response maintains its focus on ISGs, with IFIT1, IFIT2, IFIT3, MX1, and OAS2 all remaining prominently expressed. CXCL10 continues to signal for immune cell recruitment, and the addition of IFITM1 and CCL5 underscores the ongoing antiviral and immune-activating state of the airway tissues.

For MPV at 72 hours, the transcriptional landscape shifts, highlighting IL17A and an array of genes involved in cellular signaling and immune responses, such as MAP3K7, MAP3K5, and PAK1. This suggests a complex interplay between inflammatory responses and cellular reprogramming, potentially reflecting the progression of the immune response and adaptation to the viral challenge.

Finally, at 72 hours post-PIV3 infection, the transcriptional response remains robust and interferon-centric, with continued expression of IFIT1, IFIT2, IFIT3, MX1, and OAS2. The inclusion of genes like HERC5 and DDX58 at this time point suggests a mature and comprehensive antiviral state, actively engaging multiple facets of the cellular defense mechanisms to combat the viral invasion.

In summary, the OTEs showcase a strong and rapid interferon-mediated response to viral infections, adapting over time to sustain defense mechanisms, recruit immune cells, and potentially engage adaptive immune components. At 24 hours post-infection, all three viruses elicit a pronounced interferon-mediated cellular response, indicative of the body’s initial universal defense mechanism. By the 72-hour mark, while responses to IAV and PIV3 continue to heavily rely on interferon signaling, the cellular reaction to MPV exhibits a marked transformation, hinting at a sophisticated cellular approach to tackle the prolonged presence of the virus. The MAS-selected genes, thus, offer pivotal insights into the dynamic and evolving cellular defense strategies deployed against diverse viral invasions.

### 4.2. The statistically significant genes that agree when a specific virus infected OTEs are contrasted against the Mock and UV infected controls

In **Fig 3A**, the genomic intricacies of OTEs under various viral exposures are meticulously delineated, with UV and Mock treatments serving as baseline contrasts against live infections by IAV, MPV, and PIV3 at 24- and 72-hours post-infection. Beginning with IAV, a striking 212 genes were identified as common between UV-24 hour and Mock-24 hour when compared to IAV-active-24. Notable among these are IFIT2, RSAD2, and IFIT3, recognized for their antiviral efficacy, hinting at a universally conserved host reaction to IAV irrespective of its virulence. By the 72-hour mark, the shared gene count, contrasting UV and Mock against IAV-active-72, rises to 243, spotlighting crucial antiviral entities such as CXCL10 and IFITM1. This trend underscores a heightened coordinated response, potentially adapting to IAV’s dynamic impact.

Exploring MPV, 12 genes were shared between UV-24 hour and Mock-24 hour when compared to MPV-active-24. Included in this set are critical genes like ISG15 and HERC5, indicating a potentially universal defense tactic early in infection. However, by 72 hours, the shared genes reduced to a single entity in comparison to MPV-active-72, emphasizing a fluid response and the distinct challenges presented by MPV as the infection progresses. Shifting to PIV3, 10 genes were shared between UV-24 hour and Mock-24 hour against PIV3-active-24, with defensive mainstays like IFIT1 and MX1 taking the spotlight. This commonality broadens by the 72-hour point, revealing 150 shared genes when compared to PIV3-active-72, featuring genes like OAS3 and DDX58. This surge suggests a synchronized defense strategy by OTEs as the PIV3 infection advances.

While our primary emphasis lies with shared genes, unique genes also weave an intriguing story. For instance, against IAV at 24 hours, genes like NCF1 and IL27 are distinctive to UV, while the Mock exposure is marked by genes like OSM and IL9. As we approach the 72-hour mark, UV uniquely gravitates towards genes like IL12A, whereas Mock distinguishes itself with genes such as IL9 and IRF9. These distinct genes shed light on the specialized pathways OTEs might employ amidst varying viral scenarios.

In **Fig 3B**, at the 24-hour mark, the comparison of gene expression between IAV-UV-24 and IAV-active-24 against Mock-24 and IAV-active-24 highlighted 212 commonly significant genes, indicating robust overlapping gene expression alterations. The agreement percentage, a metric of expression pattern similarity in terms of log-fold change direction, stood at 88.21%. Moreover, 44 of these genes, including LAG3, CXCL10, and IFITM1, were pinpointed as top genes according to the MAS-Score. In a parallel observation, the MPV-UV-24 and MPV-active-24 compared to Mock-24 and MPV-active-24 demonstrated a flawless gene expression overlap, evidenced by a 100% agreement percentage. Twelve genes were deemed significant, with standout genes like EIF2AK2 and IFIT2. Similarly, the juxtaposition of PIV3-UV-24 and PIV3-active-24 with Mock-24 and PIV3-active-24 yielded a 100% agreement, featuring 10 mutual significant genes, including EIF2AK2 and MX1.

Progressing to 72 hours, the IAV-UV-72 and IAV-active-72 compared to Mock-72 and IAV-active-72 unveiled an 82.30% agreement. While this suggests a prevailing similarity, variations in gene expression evolution become evident. This analysis pinpointed 243 shared genes, with 42 heralded as top genes, including LAG3, CXCL10, and TAP1. Conversely, the MPV-UV-72 and MPV-active-72 comparison against Mock-72 and MPV-active-72 revealed a perfect match in gene expression alterations (100% agreement), yet, intriguingly, IRF7 was the sole gene recognized as both significant and a top gene. Finally, the PIV3-UV-72 and PIV3-active-72 comparison against Mock-72 and PIV3-active-72 observed an 80.67% agreement with 150 shared genes. Notably, 47 of these were top-tier genes, encompassing LAG3, CXCL10, and STAT1.

### 4.3. Identifying universal and unique genomic responses to viral infections

In **Fig 4**, our comparative analysis reveals intricate patterns of gene expression across the experimental conditions. The gene expression profiles at 24- and 72-hours post-infection exhibit dynamic shifts in both unique and overlapping genes across IAV-active, MPV-active, and PIV3-active conditions. At 24 hours, IAV-active stands out with 156 unique genes, followed by MPV-active with 67, and PIV3-active with 41. However, by 72 hours, PIV3-active presents an unexpected increase in unique gene count to 75, while IAV-active and MPV-active display substantial decreases to 42 and 25, respectively. This shift might hint at differential gene regulation or response mechanisms as the infection progresses.

Regarding gene overlaps, while all three conditions share 89 genes at 24 hours, this core set expands remarkably to 230 genes by 72 hours. This growth suggests an increasing commonality in the gene expression response across the conditions with time. However, examining the pairwise overlaps, the interplay becomes more intricate: while IAV-active and MPV-active’s overlap decreases, both the IAV-active & PIV3-active and MPV-active & PIV3-active overlaps expand by 72 hours.

**Fig 6** reveals that at 24 hours post-infection, distinct genetic signatures emerge, reflecting the unique and shared responses of the host to IAV and PIV3. IAV elicits a substantial immune response, influencing 220 genes, with key players such as IFNL1, IFNL2/3, and CCL5, showcasing a robust induction of interferon-stimulated genes and cytokines. Intriguingly, the appearance of CD274 suggests a potential modulation of immune checkpoints, possibly serving as a nuanced strategy to balance immune activation. Conversely, PIV3 affects a distinct and smaller set of 70 genes, highlighting IL18RAP, BPI, and IL27, which indicates a more subtle approach centered on interleukin signaling and acute phase responses. Despite these contrasting responses, there is a significant overlap in the genetic response to the two viruses, with 136 genes commonly affected. This shared response is dominated by interferon-stimulated genes such as IFIT2 and RSAD2, underscoring a collective activation of primary host defense mechanisms.

At the 72-hour mark, the landscape shifts, offering a glimpse into the prolonged and evolving host-virus interactions. IAV’s unique influence decreases to 75 genes, yet it marks a transition towards pro-inflammatory and oxidative stress responses with genes like TNF and VCAM1 taking the lead. This could be indicative of an amplified defense mechanism or a response to increased viral challenge. PIV3, in contrast, ramps up its unique influence, affecting 131 genes, with IL17A and CSF2RB at the forefront, signaling an intensified immune modulation and engagement with cytokine signaling and T-cell activation pathways. Overlapping genes between the two viral infections surge to 426, with IFIT1 and CXCL10 highly ranked, suggesting a state of sustained immune activation and highlighting the complexity of host responses as the infection progresses.

In summary, the comparative genomic analysis of host responses to IAV and PIV3 at both 24- and 72-hours post-infection reveals a complex and evolving landscape of immune modulation and cellular response. The distinct profiles of unique and overlapping genes between these two viral infections highlight the nuanced ways in which different viruses interact with and manipulate the host cellular machinery.

IAV demonstrates a potent ability to trigger a strong interferon and pro-inflammatory response in the early stages of infection, as evident from the unique and top-ranking genes at 24 hours. This robust activation of immune pathways is likely a double-edged sword, providing an initial advantage to the host in controlling viral replication, but potentially also contributing to the severe pathology often associated with IAV infections. By 72 hours, the unique IAV-induced gene profile shifts, reflecting a sustained inflammatory response and possibly the initiation of tissue repair and resolution mechanisms. Understanding how these processes are balanced and regulated is crucial for developing therapeutic strategies to mitigate the detrimental effects of excessive inflammation during IAV infection.

In contrast, PIV3 elicits a more subdued early response, with a smaller set of unique genes affected at 24 hours. However, by 72 hours, the number of unique PIV3-induced genes increases, indicating a delayed but substantial genomic response. This could reflect a stealthier strategy by PIV3, initially avoiding strong activation of the host immune response, but eventually establishing a robust interaction with the host. Deciphering the timing and nature of these interactions may provide insights into the generally milder disease course associated with PIV3 infections compared to IAV.

The substantial set of overlapping genes between IAV and PIV3, particularly prominent at 72 hours, underscores the shared challenges faced by host cells during viral infections and highlights the common pathways activated in response. This shared response likely represents a universal strategy of the host immune system to combat viral infections, and understanding these mechanisms could pave the way for broad-spectrum antiviral therapies.

Moving forward, a more comprehensive analysis, possibly incorporating additional time points and functional assays, would be invaluable in unraveling the intricate host-virus dynamics at play. The data presented here lays a solid foundation for such investigations, providing critical insights into the temporal evolution of host responses and setting the stage for future research aimed at improving our understanding and treatment of viral infections.

Moreover, when we analyze the genomic response to PIV3 infection at two distinct time points, 24- and 72-hours post-infection, using the provided network analysis given in **Fig 6B, 6C**, a shift in the host’s response becomes apparent. At 24 hours post-infection, the network is characterized by high density and connectivity among the 136 genes identified, with 45 of them showing a very strong correlation (Spearman correlation > 0.9) with at least 10 other genes. Key regulatory genes such as IFI16, IFITM1, and SP100 show exceptionally high degrees of connectivity, indicating their central role in the immediate host response to the infection. This suggests an acute and highly coordinated genomic response, possibly aimed at rapidly mobilizing the host’s defense mechanisms.

By 72 hours post-infection, however, the landscape has changed considerably. Although the total number of genes identified has increased to 426, suggesting a broadening of the genomic response, the network has become less dense, and fewer genes meet the high-correlation criteria (46 genes). Notably, the genes that dominate in terms of connectivity at this stage are different, with IFNL2/3, IFNL1, and HLA-B being the most connected. This indicates a shift in the host’s strategy, potentially moving from an immediate and generalized response to a more nuanced and targeted approach, possibly focusing on modulating the immune response and initiating repair mechanisms. **Fig 7** demonstrates the efficiency of the MAS algorithm (Algorithm 1 in **S1** File). By utilizing only the top 3 genes selected by MAS, we were able to clearly distinguish between all infection conditions and the Mock OTEs.

To bolster the findings of our study on the unique and shared genomic responses to viral infections, we draw upon the insightful research by Zissler et al. [31], which deeply explores the antagonistic regulation of key cytokines, specifically interferon-gamma (IFN-g) and interleukin-4 (IL-4), in airway epithelial cells. Their study provides a profound understanding of how these cytokines modulate gene expression in a manner that reflects the Th1 and Th2 cell differentiation pathways, which are essential for immune responses. This is particularly relevant to our observations of the interferon-stimulated gene (ISG) responses and the distinct cytokine profiles elicited by different viral infections in our OTE models.

Zissler et al. [31] demonstrated that IFN-g and IL-4 induce significant transcriptional changes in normal human bronchial epithelial cells, highlighting a complex network of gene regulation that directly antagonizes the expression pathways of each other. This mechanism mirrors the differential gene expression patterns we observed, where certain genes such as IFIT2, RSAD2, and MX1 were prominently upregulated in response to viral infections, particularly with IAV and PIV3, reflecting a strong ISG response. Furthermore, the study highlighted that IL-4 could downregulate IFN-g mediated responses, which aligns with our findings at the 72-hour post-infection mark where a shift towards more complex immune responses was noted, particularly with MPV showing a nuanced interplay between inflammatory and immune regulatory pathways.

Additionally, the identification of IL-24 as a potential biomarker for Th2 polarized conditions in Zissler et al. [31]’s study adds depth to our understanding of how epithelial cells might contribute to immune responses during viral infections. The modulation of IL-24 by IL-4 and IFN-g in their study provides a critical link to the cytokine-driven dynamics we observed in our transcriptional profiles post-infection. This supports our observations of cytokine-induced changes and suggests potential pathways through which viral infections could influence airway inflammation and immune regulation.

By integrating the insights from Zissler et al. [31] with our findings, we can better understand the underlying mechanisms of virus-induced gene expression changes and their implications for disease progression and therapeutic interventions. The parallels between the cytokine-driven gene regulation in bronchial epithelial cells and the transcriptional responses to viral infections in our OTE models underscore the relevance of our study’s outcomes in revealing both unique and universal aspects of host defense mechanisms against viral pathogens. This integration not only strengthens the validity of our identified genes but also highlights the potential for these gene signatures to inform the development of targeted treatments that leverage the antagonistic and synergistic pathways influenced by key cytokines in the airway epithelium.

Erb et al. [32]’s study provides comprehensive insights into the differential influences of type-I, type-II, and type-III interferons (IFNs) on airway epithelial integrity and their distinct genomic expressions, which correlate significantly with our observations of the interferon-stimulated gene (ISG) responses during viral infections in our OTE models.

Erb et al. [32] demonstrated that all types of IFNs induced a core set of ISGs, such as OAS1, OAS2, and IFIT2, akin to the pronounced ISG response we observed in OTEs infected with IAV and PIV3, characterized by the significant upregulation of similar genes including IFIT2, OAS2, and MX1. This underscores the fundamental role of ISGs in orchestrating an antiviral state across different types of epithelial cells and under varied IFN stimulations.

Furthermore, their findings that type-I IFNs predominantly induced genes related to cell-cell adhesion and tight junctions, while type-III IFNs favored genes important for transepithelial transport, help explain the varied transcriptional landscapes we noted at different post-infection times and under different viral challenges. For instance, our observation of robust and dynamic ISG expressions such as IFIT1, IFIT2, IFIT3, and the OAS family across the first 24 to 72 hours post-infection could reflect a similar underlying mechanism of IFN-induced epithelial fortification and immune signaling modulation as described by Erb et al. [32].

Their study also highlighted how type-II IFN promoted pro-inflammatory genes and genes associated with cell proliferation, which aligns with the increased expression of inflammatory and cell signaling genes such as IL17A and various MAP kinases in our MPV-infected OTEs by 72 hours. This suggests a strategic shift in the epithelial response to prolonged viral presence, potentially mirroring the differential IFN responses that orchestrate either a barrier-enhancing or an inflammatory outcome based on the IFN type and the epithelial context.

By integrating Erb et al. [32]’s insights into our analysis, we can more deeply appreciate the complexity of IFN responses in epithelial cells exposed to viral infections. The common and unique gene expressions identified in both studies not only validate our findings but also enhance our understanding of how different IFNs tailor epithelial defense mechanisms against viral infections. This comparative analysis lends robust support to the notion that while the immediate ISG response forms the first line of defense against viral replication and spread, the subsequent epithelial responses, influenced by different IFNs, are crucial in shaping the trajectory of immune reactions and cellular recovery processes post-infection.

In our study, the significant upregulation of interferon-stimulated genes (ISGs) such as IFIT1, IFIT2, IFIT3, and MX1 in response to IAV and PIV3 infections aligns with findings by Jakwerth et al. [33], who report a broad and strong upregulation of genes related to the kinin-kallikrein system (KKS) in the context of SARS-CoV-2 infection. The activation of these pathways underscores a common strategy within epithelial cells to counteract viral infections through modulation of immune responses and cellular signaling processes. Notably, the kinin-kallikrein system, implicated in the pathophysiology of COVID-19 by Jakwerth et al. [33], parallels our observations of a robust antiviral state mediated by ISGs, suggesting a universal epithelial response to viral threats that includes both rapid ISG upregulation and modulation of receptor-mediated signaling pathways. This comparison not only validates the significance of the MAS-selected genes identified in our analysis but also provides a broader context for understanding the cellular mechanisms that underpin resistance to viral infections across different viruses, including SARS-CoV-2.

## 5. Conclusions

Our study presented a comprehensive comparative analysis of the host’s transcriptional response to three distinct viral infections (IAV, MPV, and PIV3) in OTEs at 24- and 72-hour time points. We employed the Magnitude-Altitude Score (MAS) algorithm to unveil the intricate gene expression dynamics, revealing both unique and shared pathways activated in response to these viral challenges. In the context of IAV infection at 24 hours, a strong interferon-stimulated gene (ISG) response is evident, with significant upregulation of IFIT2, RSAD2, IFIT3, IFNL1, IFIT1, IFNB1, ISG15, OAS2, OASL, and MX1. These genes collectively orchestrate a robust antiviral defense in airway tissues. RSAD2 and the OAS family reinforce the antiviral state, engaging in vital viral component degradation. MPV infection at 24 hours showcases a nuanced response, encompassing innate and adaptive immune signals. Highly expressed ISGs such as IFIT1, IFIT2, IFIT3, RSAD2, MX1, and OAS2 maintain a potent antiviral state. CXCL10’s upregulation indicates immune cell recruitment, while the presence of TXK and CD79A suggests early adaptive immune engagement. PIV3 infection at 24 hours triggers a transcriptional response dominated by ISGs like IFIT1, IFIT2, IFIT3, MX1, and OAS2, reflecting a potent antiviral state. CXCL10 signals active immune cell recruitment, and OSM likely contributes to inflammation modulation. Genes RSAD2 and HERC5 highlight the comprehensive cellular response to PIV3 infection.

At 72 hours post-IAV infection, ISGs including IFIT1, IFIT2, IFIT3, MX1, and OAS2 remain significantly expressed, while CXCL10 persists, indicating ongoing immune cell recruitment. IFITM1 and CCL5 join the antiviral and immune-activating state. MPV infection at 72 hours reveals a transcriptional shift toward IL17A and genes related to cellular signaling and immune responses, implying a complex interplay between inflammation and cellular reprogramming. This suggests adaptation to the viral challenge over time. Finally, at 72 hours post-PIV3 infection, the transcriptional response remains interferon-centric, with continued expression of IFIT1, IFIT2, IFIT3, MX1, and OAS2. Genes like HERC5 and DDX58 indicate a mature and comprehensive antiviral state.

Analysis of frequently appearing genes highlights the pivotal role of ISGs across all infections and time points, emphasizing their universal importance in antiviral defense. Temporal shifts in gene expression profiles, particularly in MPV infection, underscore the dynamic host-virus interaction, suggesting adaptation and fine-tuning of the immune response. Additionally, the identification of shared and unique genes reveals host-tailored responses to specific pathogens. Commonalities across infections hint at universal defense mechanisms, while unique genes illuminate specialized response pathways. In response to viral infections, IAV demonstrates a robust and sustained impact on genes from the initial 24 hours, persisting up to the 72-hour mark, highlighting a profound and enduring influence marked by early inflammatory responses. In contrast, PIV3’s effect intensifies between 24 and 72 hours, reflecting a delayed yet substantial genomic response, indicative of a gradual, nuanced strategy.

## 6. Study limitations

While the 3D airway OTE model at the ALI provides a more in-depth understanding of airway biology, several limitations should be considered:

- Absence of Systemic Factors: The model does not incorporate systemic influences such as blood flow or hormonal regulation, which can affect airway physiology and response to treatments in vivo.
- Physical Constraints: The static nature of the model may not fully replicate the dynamic physical forces exerted by breathing, such as shear stress and changes in airway pressure.
- Biomechanical Properties Limitation: Although the hydrogel’s stiffness can be varied, the range may not cover all physiological conditions, particularly those relating to severe pathological states that significantly alter the airway’s mechanical properties.

These limitations suggest that while the 3D ALI model significantly enhances our understanding of airway biology, results should be interpreted with an understanding of its constraints relative to the full complexity of the human respiratory system. Additionally, the study’s focus on only two time points (24- and 72-hours post-infection) may overlook crucial changes in host-virus interactions happening at different intervals, potentially missing nuances in the temporal evolution of the response. These limitations emphasize the need for complementary in vivo studies and finer temporal resolution to enhance the study’s applicability and depth of understanding.

## Supporting information

S1 File Supporting information for exploring the host response in infected lung organoids using NanoString technology: A statistical analysis of gene expression data. Algorithms.
(DOCX)
