# Supplementary material for: Exploring the host response in infected lung organoids using NanoString technology: A statistical analysis of gene expression data
Source: PLoS One. 2024 Nov 26;19(11):e0308849. doi: 10.1371/journal.pone.0308849 (PMC11594423; doi:10.1371/journal.pone.0308849)
Supplement: S1 File — Algorithms. (DOCX) [file pone.0308849.s001.docx]

**Supporting Information**

Exploring the Host Response in Infected Lung Organoids Using NanoString Technology: A Statistical Analysis of Gene Expression Data

Mostafa Rezapour, PhD^1*^; Stephen J. Walker, PhD ^2^; David A. Ornelles, PhD ^3^; Muhammad Khalid Khan Niazi, PhD ^1^; Patrick M. McNutt, PhD ^2^; Anthony Atala, MD ^2^; Metin Nafi Gurcan, PhD ^1^

^1^Center for Artificial Intelligence Research, Wake Forest University School of Medicine, Winston-Salem, NC, USA

^2^ Wake Forest Institute for Regenerative Medicine, Wake Forest University School of Medicine, Winston-Salem, NC, USA

^3^ Department of Microbiology and Immunology, Wake Forest University School of Medicine, Winston-Salem, NC, USA

*** Correspondence:**Mostafa Rezapour, PhD
[mrezapou@wakehealth.edu](mailto:mrezapou@wakehealth.edu)

**Algorithms**

| **Algorithm 1.** **Magnitude-Altitude Score (MAS)** |
| --- |

**Input**: Given r gene expressions for Baseline Condition (e.g., Mock-24) and Treated Condition (e.g., IAV-None-24).

**Step 1:** Perform two-sample independent t-test (α=0.05) [1], and calculate p-value denoted by $p_{i}$, for $i=1, 2, \ldots, r.$

**Step 2:** Adjust p-values using the Banjamini-Hochberg (BH) method [2]:

**Step 2.1:** Sort the p-values in ascending order and assign a rank from 1 to r.

**Step 2.2:** Set $p_{i}^{B}=\frac{{mp}_{i}}{i},$ for $i=1, 2, \ldots, m,$where $p_{i}$ is the p-value corresponding to gene with rank $i$ and $m$ is the number of tests.

**Step 2.2:** Set $k=m$, and $p_{k}^{BH}=p_{k}^{B}$

**Step 2.3:** While k$>1$, do:

**Step 2.3.1:** If $p_{k}^{BH}\leq$1, then go to the next step. Otherwise, set $p_{k}^{BH}$=1.

**Step 2.3.2:** If $p_{k}^{\mathrm{BH}}>p_{k+1}^{\mathrm{BH}}$, then set $p_{k}^{BH}=p_{k+1}^{BH}$.

**Step 2.3.3:** Decrement $k$, i.e., $k=k-1$.

**Step 2.3.4:** Return $p_{k}^{\mathrm{BH}}$ for $k=1, 2, \ldots, m$.

**Step 3**: Compute the MAS score only for genes whose null hypotheses are rejected based on the BH-adjusted method ($p_{l}^{BH}<\alpha)$.

**Step 3.1**: Compute$\text{ log2FC}_{l}=\log_{2}(\frac{{\mathrm{Average}\left( A \right)}_{l}}{{\mathrm{Average}\left( B \right)}_{l}}),$ for l$=1, 2, \ldots, s,$where $s$ is the number of rejected null hypotheses rejected based BH adjusted method ($p_{l}^{BH}<\alpha)$. Moreover, ${\mathrm{Average}\left( A \right)}_{l}$and ${\mathrm{Average}\left( B \right)}_{l}$represent the average of gene $l$ for replicates in the Baseline and Treated conditions, respectively.

**Step 3.2:**  Compute MAS score, $\text{MAS}_{l}=$| $\text{(log2FC}_{l})|^{M}|{(\log_{10}(p}_{l}^{\mathrm{BH}})) |^{A}$, for l$=1, 2, \ldots, s$, where $M$ and $A$ are hyper-parameters.

**Step 3.3:**  Sort genes in terms of $\text{MAS}_{l}$ in a descending order and rank the gene with largest MAS as rank 1, and the gene with smallest MAS score as rank s.

**Output:** Return BH significant genes, their MAS ranks, and MAS score of BH significant genes: $G,R$ and $\Upsilon$.

____________________________________________________________________________________

| **Algorithm 2.** **Agreement Assessment of Common Genes with Dual Baselines** |
| --- |

**Input**: Gene expressions for

- $\mathrm{Baseline}_{T_{i}}$: Control group at time $T_{i}$, for $i=1, 2, ..., t.$
- ${Negative Control}_{T_{i}}$: Negative control group at time $T_{i}$, for $i=1, 2, ..., t.$
- $\mathrm{Treated}_{T_{i}}^{X}$: Group of OTEs infected by the $X^{th}$ virus at time $T_{i}$, for $X=1, 2, ..., q,$ and $i=1, 2, ..., t.$
- $n_{T_{i}}^{X}$=0.
- $A_{T_{i}}^{X}$={}.

**Step 1:** **For** $i=1$ to $t$:

**For** $X=1$ to $q$:

- ${(G_{T_{i}}^{X},R}_{T_{i}}^{X}, \Upsilon_{T_{i}}^{X},\Xi_{T_{i}}^{X},P_{T_{i}}^{X})$= MAS ($\mathrm{Baseline}_{T_{i}}$, $\mathrm{Treated}_{T_{i}}^{X}$, M=1, A=1),
- ${({G^{'}}_{T_{i}}^{X},R'}_{T_{i}}^{X}, \Upsilon_{T_{i}}^{'X},{\Xi'}_{T_{i}}^{X},{P'}_{T_{i}}^{X})$= MAS (${Negative Control}_{T_{i}}$, $\mathrm{Treated}_{T_{i}}^{X}$, M=1, A=1),
- ${G"}_{T_{i}}^{X}=G_{T_{i}}^{X}\cap{G^{'}}_{T_{i}}^{X}$.

**End (For)**

**End (For)**

**Step 2:** **For** $i=1$ to $t$:

**For** $X=1$ to $q$:

**If** ${G"}_{T_{i}}^{X}\neq\emptyset$:

**For** each gene in the common set $g\epsilon{G"}_{T_{i}}^{X}$:

- Retrieve the $\text{log2FC}$ values from both comparisons: $\overset{\begin{aligned} Entry \\ for gene g \end{aligned}}{\overbrace{\Xi_{T_{i}}^{X}}}$and $\overset{\begin{aligned} Entry \\ for gene g \end{aligned}}{\overbrace{{\Xi'}_{T_{i}}^{X}}}$.
- If $\overset{\begin{aligned} Entry \\ for gene g \end{aligned}}{\overbrace{\Xi_{T_{i}}^{X}}}$and $\overset{\begin{aligned} Entry \\ for gene g \end{aligned}}{\overbrace{{\Xi'}_{T_{i}}^{X}}}$ have the same sign, increment agreement count, $n_{T_{i}}^{X}$=$n_{T_{i}}^{X}$+1, and $A_{T_{i}}^{X}=A_{T_{i}}^{X}\cup g.$

**End (For)**

**End (For)**

**End (For)**

**Step 3:** **For** $i=1$ to $t$:

**For** $X=1$ to $q$:

Calculate Agreement Ratio:

${AR}_{T_{i}}^{X}=$ $\frac{n_{T_{i}}^{X}}{\left| {G”}_{T_{i}}^{X} \right|}\times100,$

where $\left| {G”}_{T_{i}}^{X} \right|$ is the number of genes in the common set.

**End (For)**

**End (For)**

**Output:** $A_{T_{i}}^{X}$ and ${AR}_{T_{i}}^{X}$for $i=1, 2, \ldots, t$, and $X=1, 2, \ldots, q.$

____________________________________________________________________________________

| **Algorithm 3.** **Common genes selection** |
| --- |

**Input**: Gene expressions for

- $\mathrm{Baseline}_{T_{i}}$: Control group at time $T_{i}$, for $i=1, 2, ..., t.$
- $\mathrm{Treated}_{T_{i}}^{X}$: Group of OTEs infected by the $X^{th}$ virus at time $T_{i}$, for $X=1, 2, ..., q,$ and $i=1, 2, ..., t.$

**Step 1:** Identify commonly BH-Significant genes at each time:

**For** $i=1$ to $t$:

$G_{W_{i}}=\{\}$.

**For** $X=1$ to $q$:

- ${(G_{T_{i}}^{X}R}_{T_{i}}^{X}, \Upsilon_{T_{i}}^{X},\Xi_{T_{i}}^{X},P_{T_{i}}^{X})$= MAS ($\mathrm{Baseline}_{T_{i}}$, $\mathrm{Treated}_{T_{i}}^{X}$, M=1, A=1),
- $G_{W_{i}}=G_{W_{i}}\cup{\{G}_{T_{i}}^{X}\}$.

**End (For)**

Set MAS-Common-genes-$T_{i}$= $\bigcap_{X=1}^{q} G_{T_{i}}^{X}$.

**End (For)**

**Output:** MAS-Common-genes-$T_{i}$

____________________________________________________________________________________

| **Algorithm 4.** **Network-Based Gene Correlation Analysis** |
| --- |

**Input**:

- Import the gene expression dataset, ensuring that it is organized in a tabular and structured format suitable for analysis. Assume that the $i^{th}$column of the data, denoted as$v_{i}$, represents a vector containing the expression levels of the $i^{th}$ gene in the list of genes for $\mathrm{Baseline}_{T_{i}}$, and $\mathrm{Treated}_{T_{i}}^{X} \mathrm{for}X=1, 2, ..., q.$
- Set $M=20,$ and $\rho=0.8$.

**Step 1:** Spearman Correlation Matrix Computation [3]:

- Calculate the pairwise Spearman correlation for all genes in the dataset.
- Identify and retain correlations that are above the threshold $\rho$ in magnitude, ensuring only strong and significant interactions are considered for network construction. Discard the rest.

**Step 2:** Network Construction:

- Construct a network where nodes represent genes, and edges represent significant correlations between genes.
- For each significant correlation identified in Step 1, create an edge between the corresponding genes in the network.

**Step 3:** Gene Selection Based on Degree:

- Calculate the degree (number of edges) of each gene in the network.
- Filter out genes that have a degree less than M, ensuring that only genes with a sufficient number of significant interactions are included in the final network.

**Output:** Visualize the resulting gene network

____________________________________________________________________________________

| **Algorithm 5.** **Unique gene selection for a particular virus** |
| --- |

**Input**: Gene expressions for

- $\mathrm{Baseline}_{T_{i}}$: Control group at time $T_{i}$, for $i=1, 2, ..., t.$
- $\mathrm{Treated}_{T_{i}}^{X}$: Group of OTEs infected by the $X^{th}$ virus at time $T_{i}$, for $X=1, 2, ..., q,$ and $i=1, 2, ..., t.$

**Step 1:** **For** $i=1$ to $t$:

$G_{T_{i}}^{X}=\{\}$,

**For** j$=1$ to $t (j\neq i)$:

- - $G_{T_{i}}^{unique}$= $G_{T_{i}}^{unique}\cup$ (MAS-Common-genes-$T_{i}$ \ MAS-Common-genes-$T_{j}$), where “\” is the set difference operation. Let $\tau_{i}^{'}$ be the cardinality of MAS-Unique-to-$T_{i}$, $G_{T_{i}}^{unique}$.

**End (For)**

**End (For)**

**Output:** $G_{T_{i}}^{X}$ for $i=1, 2, \ldots, t$, and $X=1, 2, \ldots, q.$

____________________________________________________________________________________

# **References**

| [1] | T. K. Kim, "T test as a parametric statistic," *Korean journal of anesthesiology,* vol. 68, no. 6, pp. 540-546, 2015. |
| --- | --- |
| [2] | Benjamini, Yoav and Hochberg, Yosef, "Controlling the false discovery rate: a practical and powerful approach to multiple testing," *Journal of the Royal statistical society: series B (Methodological),* vol. 57, no. 1, pp. 289-300, 1995. |
| [3] | Hauke, Jan and Kossowski, Tomasz, "Comparison of values of Pearson's and Spearman's correlation coefficients on the same sets of data," *Quaestiones geographicae,* vol. 30, no. 2, pp. 87-93, 2011. |
